# Microbial habitability of Europa sustained by radioactive sources

**DOI:** 10.1038/s41598-017-18470-z

**Published:** 2018-01-10

**Authors:** Thiago Altair, Marcio G. B. de Avellar, Fabio Rodrigues, Douglas Galante

**Affiliations:** 1 Brazilian Synchrotron Light Laboratory (LNLS), Brazilian Center for Research in Energy and Materials (CNPEM)., Av. Giuseppe Máximo Scolfaro, 10000, 13083-100 Campinas, SP Brazil; 2 Instituto de Astronomia, Geofísica e Ciências Atmosféricas, Universidade de São Paulo., Rua do Matão, 1226, 05508-090 São Paulo, SP Brazil; 3 Departamento de Química Fundamental Instituto de Química, Universidade de São Paulo., Av. Prof. Lineu Prestes, 748, 05508-000 São Paulo, SP Brazil; 4 Programa de Pós-Graduação em Física Biomolecular, Instituto de Física de São Carlos, Universidade de São Paulo, São Carlos, SP Brazil

**Keywords:** Microbial ecology, Astrobiology

## Abstract

There is an increasing interest in the icy moons of the Solar System due to their potential habitability and as targets for future exploratory missions, which include astrobiological goals. Several studies have reported new results describing the details of these moons’ geological settings; however, there is still a lack of information regarding the deep subsurface environment of the moons. The purpose of this article is to evaluate the microbial habitability of Europa constrained by terrestrial analogue environments and sustained by radioactive energy provided by natural unstable isotopes. The geological scenarios are based on known deep environments on Earth, and the bacterial ecosystem is based on a sulfate-reducing bacterial ecosystem found 2.8 km below the surface in a basin in South Africa. The results show the possibility of maintaining the modeled ecosystem based on the proposed scenarios and provides directions for future models and exploration missions for a more complete evaluation of the habitability of Europa and of icy moons in general.

## Introduction

Except for Earth, the moons of the giant planets, especially the icy moons such as Enceladus, Europa, Ganymede and Callisto, are the only other places in the Solar System with considerable evidence of the existence of liquid water in abundance. The oceans under the icy crusts on these moons may be able to host living organisms^1–8^ and thus be able to host habitable environments. Two critical factors that can make these moons compelling environments for living systems, besides the presence of water, are the availability of energy and a chemical disequilibrium based on reductant-oxidant pairs f or biological processes^3^. The surface of the Jovian moon Europa is young, with a resurfacing age determined to be approximately 30–70 Myr^9^, and it represents one of the most important targets for astrobiology research in the Solar System^10^. Studies also indicate active geological processes on a global scale on this icy moon such as the movement of lithospheric blocks and the upwelling of material that fills the rocky core^3,11,12^. These aspects could help to maintain the chemical disequilibrium in the oceans under its icy layer, which is powered by tidal and radiolysis phenomena (the latter is emphasized in this paper).

Radiolysis has already been proposed for the Europan framework. However, it was mainly focused on radiolysis caused by the bombardment of the superficial ice with energetic charged particles, such as electrons or ions, that are accelerated by the magnetic field of a nearby planet (in this case, Jupiter)^3,13^. Another possibility for the radiolysis on Europa comes from the bottom of the ocean, where there is a water-rock interface. Radioactive decay also occurs from fissionable materials that exist in every rocky celestial body in the Solar System. These materials, primordial radioactive elements, emit ionizing radiation that can interact with the ocean water that breaks its molecules, causes excitation and ionization, and consequently and locally forms very reactive ionized or radical species^4,14–17^. This defines the water radiolysis, the focus of this work. Research on the radiation effects on water and mainly radical formation induced by radiolysis increased largely in the mid-20^th^ century^14^; however, the association of radiation from nuclear decay as a possible source of energy for a living system was proposed near the end of the century^18^. Recent studies of the so-called fossil natural reactors on Earth^18^ have provided a basis for the debate on the importance of ionizing energy from radioactive decay as a localized source of energy for biological processes. Notwithstanding, the recent discovery of peculiar ecosystems in deep subsurface environments, which are maintained by nutrients produced via radionuclide radiolysis^19–21^, has garnered attention for its feasibility.

On Earth, water radiolysis is significant in the deep environments where water and fissionable materials exist^17,19,22,23^ and consequently form several chemical species that contribute to microbial activity^20,21^. Chivian *et al*.^20^ and Lin *et al*.^21^ reported an important occurrence in nature of metabolism dependent on this type of radioactivity interaction. In the depths of the Mponeng gold mine in South Africa^20,21^ and located at the region of the Witwatersrand basin, it was found that a single-species ecosystem based on the bacterium *Candidatus Desulforudis audaxviator*, which uses this source of energy, was independent of sunlight. This discovery opened new venues to the study of other non-illuminated environments of the Solar System and the Universe, including Europa and other icy moons.

Recently, the debate on radiolysis under the surface of Europa has gained new perspectives. Atri^24^ discusses the importance of galactic cosmic rays (GCR), which are primary charged particles, mostly protons, that originated beyond the Solar System. If a celestial body has a reasonably thick atmosphere, primary GCR particles strike the atmospheric molecules, producing secondary particles such as kaons, pions and muons that can propagate deep underground and are highly unstable, quickly decaying to produce particles such as β and γ particles and possibly triggering radiolysis. The radiolysis discussed in that work^24^ is galactic cosmic ray-induced and may be important when considering small rocky bodies such as planets not tied to any planetary system or comet, but it depends on the presence of an atmosphere. However, radiolysis from radioactive isotope decay has shown potential importance in powering life on the deep subsurface of icy moons where solar energy cannot reach and galactic cosmic rays cannot provide enough energy. Considering charged particles of reasonable primary energy, only muons could reach 3 km below the surface level of an icy moon, and the energy deposition rate would still become nearly zero below this depth^25,26^.

However, there is still a modest number of references in the literature related to the effect of water radiolysis as a consequence of radioactive minerals in the deep subsurface icy moons and its implications for habitability. Recently, the radiolytic production of H_2_ in the subsurface of several of the Solar System’s icy moons was proposed^13,27^, although there is a necessity for complementary models to associate radiolytic energy production with biological metabolism to assess the actual habitability of extensive extraterrestrial water bodies.

Models related to the survival of bacterial cells based on radiolysis-produced chemical species, such as H_2_, have been proposed^28^. This model focuses on terrestrial context and on the primary radiolysis product. In contrast, in this study, we present the model based on the production of a secondary chemical species, sulfate, and apply it to the extraterrestrial context. For this model, we compared the radiolysis-produced sulfate rate to *in situ* sulfate demand for a deep subsurface environment where *Ca. D. audaxviator* was found.

Thus, this paper approaches the possibility of maintaining an ecosystem based on the chemical energy provided as a consequence of the direct radiolysis of water by primordial long-living radionuclides such as ^238^U, ^232^Th and ^40^K on a modeled setting for Europa. This hypothesis was based on the importance of such species for the natural radioactivity on Earth and assuming a similar elemental distribution on rocky planets and on Europa. The potential habitability of Europa is the main reason for the choice of this moon for the model in addition to the reasonable and growing body of information on its subsurface ocean and crust^11^ as well as for its importance for future space missions such as the ESA mission JUpiter ICy moons Explorer (JUICE)^10^. It was possible to show the availability, under certain conditions, of enough chemical energy to sustain an ecosystem of chemoautotrophic extremophilic candidate species such as *Ca. D. audaxviator*, which has a metabolism based on sulfur reduction. Such an energy source could be used to sustain basic metabolism and/or repair damage caused by radiation exposure^20^, thus creating a habitable environment, from the energetic point of view. *Ca. D. audaxviator* was found in fractured water in the gold mine of Mponeng, located in the Witwatersrand basin region^20^, South Africa, surviving at depths up to 2.8 km below the surface with temperatures between 40 °C and 60 °C, a pH of 9.3, pressures comparable to those of the abyssal regions of Earth’s oceans, low availability of nutrients and some concentration of radioactive minerals such as uraninite (UO_2_)^19,20^. This work proposes the use of this microorganism as a biological model and deep terrestrial environments as analogues to better understand the limits of habitability on deep, non-illuminated environments of the Solar System, although this scenario is still poorly explored for this purpose.

## Biological Energy Transduction

**Figure 1:**
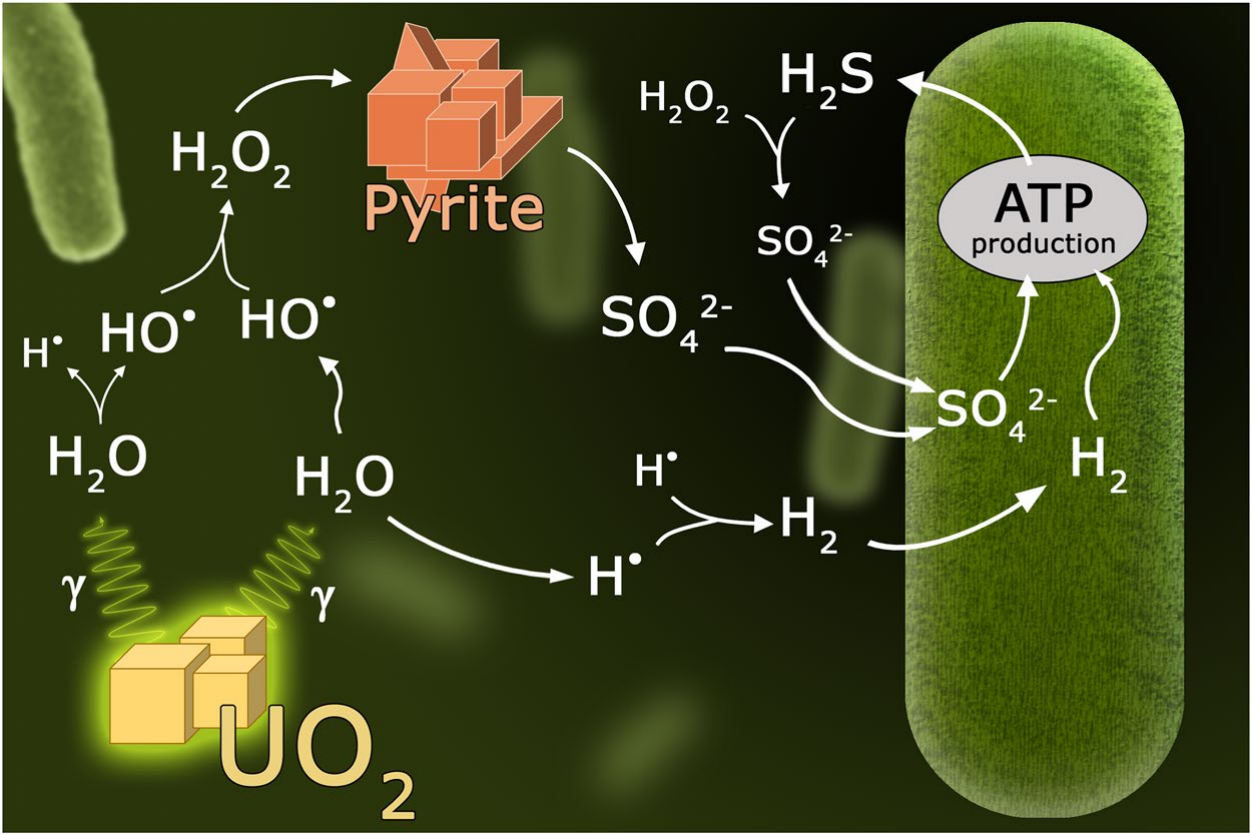
Model of the single-species ecosystem of the fractured Mponeng gold mine in South Africa. *Ca. D. audaxviator*’s pathway for obtaining energy from the decay of uranium of ^238^UO_2_ (uraninite) is shown, including the pathway of sulfate reduction. Adapted from Chivian *et al*.^20^.

Since the first examples of a single-species microbial ecosystem were reported from deep subsurface environments^20,29^, attention has been drawn to bacteria similar to *Ca. D. audaxviator* on other extreme regions. Figure 1 illustrates a simplified model of the metabolic pathway of *Ca. D. audaxviator*, which obtains energy from the radiolysis of water. The bacterium extracts energy from the sulfate $(S{O}_{4}^{-2})$ reduction reaction, as shown in reaction (1)^21^.

1 $$4{{H}}_{2}+{{H}}^{+}+{S}{{O}}_{4}^{2-}\to {H}{{S}}^{-}+4{H}_{2}{O}$$

The bacterial sulfate consumption model calculations depend on the presence of H_2_ in such a way that sulfate is the limiting reactant of reaction (1). Dissolved H_2_ may exist on icy moons such as Enceladus as a result of radiolysis or hydrothermal activity. The presence of this gas in the subsurface ocean has been reported for Enceladus^13^. On Europa, there are also speculations related to this component^27,30^.

## Physicochemical Basis For Radiolysis

As a benchmark for this work, the experimental results of Lefticariu *et al*.^17^ and Lin *et al*.^21^ were used for the calculations. The first study provided a model for the rate of radiolytic sulfate production by the exposure of water and pyrite to a source of gamma radiation. The second work presents the *in situ* rate of microbial sulfate reduction between 0.22 and 1.45 nM per year for a cell density of 4 × 10^7^ cells per liter or 5.5 × 10^–18^ to 3.6 × 10^–17^ mols per cell per year in the case of the deep subsurface of the Witwatersrand basin^20^.

The experimental work conducted by Lefticariu *et al*.^17^ showed that the sulfate production via radiolysis is *G*_*s*_ = *2.1* × *10*^*−9*^ *[mol/m*^2^*/(J/kg)]*. An identified source of $({{\rm{SO}}}_{4}^{-2})$ in this environment is the oxidation and dissolution of pyrite (FeS_2_). This mineral is known for its primordial origin on rocky planets^31^, with possible implications on prebiotic chemistry and early metabolism. It can react with a radiolysis product of water due to the radiation released by minerals such as UO_2_ or ThO_2_^32^, as expressed in reaction (2).

2 $$Fe{S}_{2}+4H{O}^{\cdot }\to F{e}^{2+}+S+S{O}_{4}^{-2}+2{H}_{2}$$

The $H{O}^{\cdot }$ radical is one of the products of the primary reactions of the radiolysis of water^32,33^ (3).

3 $${H}_{2}{O}^{\underrightarrow{IonizingRadiation}}\,{e}_{aq}^{-},{H}^{\cdot },H{O}^{\cdot },H{O}_{2}^{\cdot },{H}_{2}{O}_{2},{H}^{+},O{H}^{-},{H}_{2}$$

The production rate of $S{O}_{4}^{2-}$ as a function of the dose D_i_ of (gamma) radiation from different species is then given by Y_s_ = Σ_i_ D_i_ × G_s_ in units of *mol/m*^2^*/year*. A direct relationship between G_s_ and Y_s_ and the exposed surface area of pyrite exists, which is a feature that is explored further in section 5 and in the supplementary material. The dose D_i_ is given by *D*_*i*_ = *E*_*i*_ × *λ*_*i*_ × *c*_*i*_ × *N*_*a*_ × *A*_*i*_^*−1*^ *[J/kg/year]*, where *E [J/decay]* is the energy per decay corrected by neutrino loss *via* beta decay, *λ* = *1/T*_*1/2*_ *[decay per year]* is the decay constant (in conformity with Lefticariu^17^,), *c [ppm]* is the concentration of the radioactive element, *N*_*a*_ is Avogadro’s number, and *A*_*i*_ *[g/mol]* is the atomic mass. The index *i* stands for the radioactive species involved in the process. Finally, the annual production of radiolytic sulfate is given by *P*_*s*_ = *Y*_*S*_ × *S*_*py*_ × *w*_%_, where *S*_*py*_ *[m*^2^*/kg*_*rock*_] is the surface area of pyrite per kilogram of sedimentary rock, which, for pyrite in the authors’ experiment, was measured to be 226.0 ± 6.5 cm^2^/g, and w_%_ is the pyrite mass percentage for the rock.

## Abundances Of Radioactive Materials And Pyrite Granulometry

**Figure 2:**
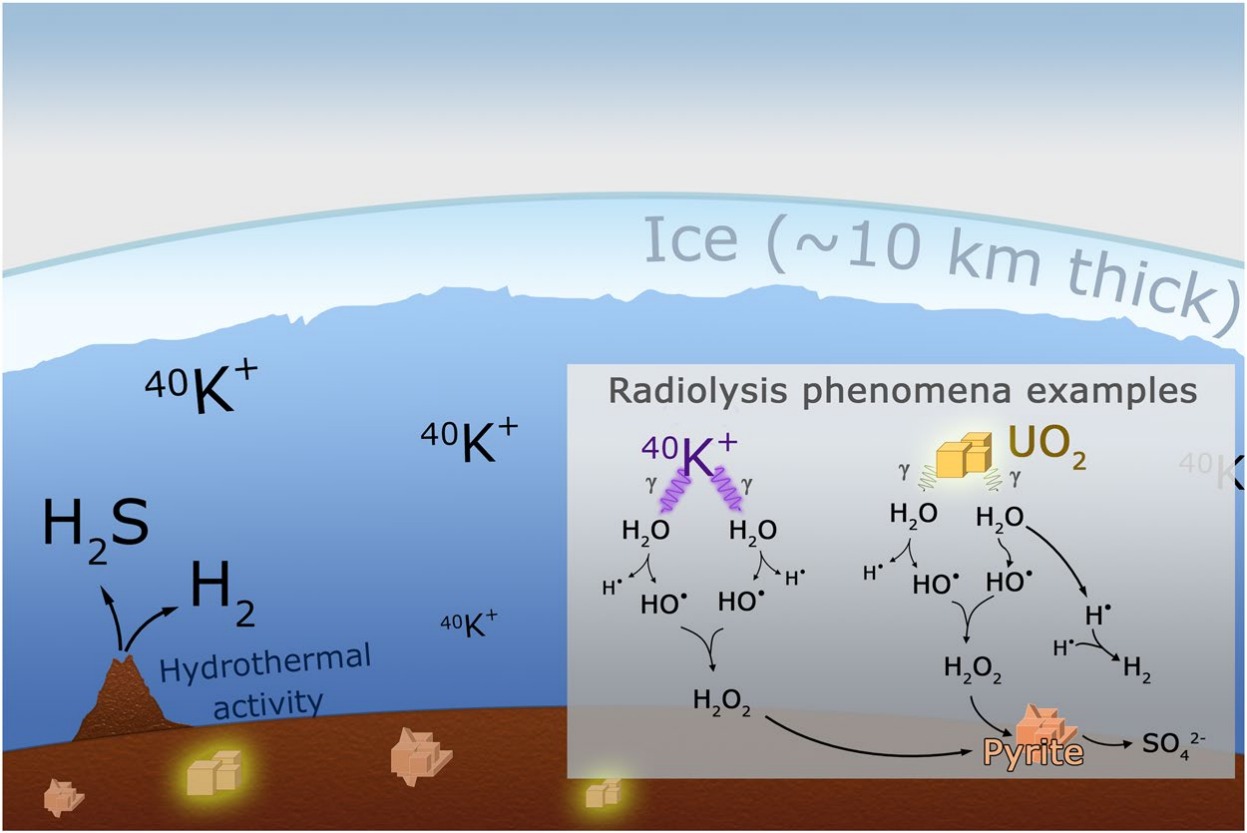
Model of Europa emphasizing the radiogenic material and the gamma-ray radiolysis phenomena occurring in the seabed. We assume ^238^U and ^232^Th are within the rocks of the Jovian moon and ^40^K is dissolved in the water. Adapted from Chyba & Hand (2001).

The results for radiolysis-produced sulfate, which was induced by natural radioactive decay in the Mponeng mine fracture water, suggests the possibility of an analogous process occurring in the subsurface environment of Europa, as schematized in Fig. 2. This work assumes that there are clumps of radioactive materials in the seabed, proximate to pyrite formations and far from hydrothermal vents that could be another source of sulfate and significantly increase the temperature of the medium. Models for the origin, composition and evolution of the crust and ocean of Europa^11^ suggest the formation of pyrite-like materials, which are a major component. It was assumed that the niches of radioactive materials contain ^238^U and ^232^Th and that ^40^K is present in the ocean in concentrations that are expected to be higher than what is found in modern terrestrial oceans^34^.

For uranium and thorium, the concentration from three subsurface scenarios^17^ was used because the actual concentrations on the Europa seabed are still unknown: a) Martian deep vadose (water-unsaturated zone); b) rocks from non-mineralized strata having low concentrations of radioactive elements from the Witwatersrand basin; and c) rocks from mineralized strata having high concentrations of radioactive elements on the Witwatersrand basin. For potassium, it was assumed that concentrations were in the range of 380 ppm (as in Earth’s ocean^34^) to 3800 ppm (which may be closer to that of the Europan ocean^8^), with the unstable ^40^K isotope^35^ accounting for 0.0117%.

For the pyrite sites, the presence of homogeneous pyrite grains covering parts of the niches was assumed. We use the experimental value of the surface area of pyrite, *S*_*py*_ = *226* *cm*^2^/*g*^17^, related to grains in the range of 100 to 150 μm in the size as a base to calculate other plausible scenarios for the granulometry that could possibly exist on Europa. These grains of pyrite were modeled as small spheres that fill a cubic space, just as a typical sphere-packing model. Varying the spherical grain size, i.e., the diameter of the sphere (φ), implies a variation of the surface area of the grain, S_py_(φ). Considering this model, the total surface area of the sphere packing is inversely proportional to the sphere radius. Thus, the sulfate production (P_s_) was estimated for different possible grain sizes based on the Wentworth^36^ grade scale, considering the concentrations of radionuclides and pyrite for the different scenarios described earlier. For each type of aggregate, a homogeneous grain size distribution was considered. In other words, every grain has the same average size.

## Results

**Figure 3:**
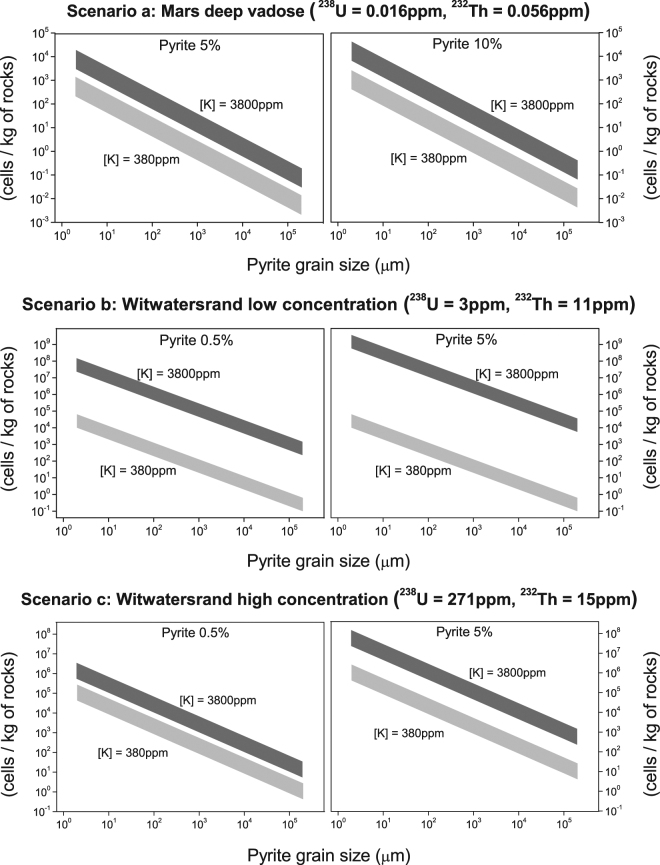
Log-Log plot of the cell-carrying capacity per mass of rocks that contains pyrite compared to the results for the different uranium and thorium scenarios (**a**,**b** and **c**, as described in section 4) and the assumed minimum (light gray) and maximum (dark gray) potassium concentrations. The X-axis represents the variation in grain size of pyrite based on the classification and based on the Wentworth scale (see Table S2), which is inversely proportional to the surface area available for oxidation.

Table 1 shows the calculated total surface area of pyrite for the different types of aggregates. Based on the values in Table 1, the sulfate production per year as a function of the pyrite rock mass (presented on Table S1) was calculated as well as the cell-carrying capacity (the quantity of cells that could survive) of *Ca. D. audaxviator* per kilogram of rock present on the local site for the proposed analogue scenarios (summarized in Fig. 3 and further described in Table S2). The radiolytic sulfate production rates were based on the model of Lefticariu *et al*.^17^, but greater values for the rates were obtained, not only due to the addition of the ^40^K source but also due to a revision of the previous calculations.

**Table 1:** Sphere-packing model results for the surface area of pyrite from different types of aggregates, based on the Wentworth scale^36^.

Type of aggregate	Grain φ(μm)	Spy(φ) (m^2^.kg^−1^)
Clay	2	1.41 × 10^3^
Silt	10	2.83 × 10^2^
	60	4.71 × 10^1^
Sand	125	2.26 × 10^1^
	500	5.65
	1000	2.83
Pebbles	10000	2.83 × 10^−1^
	50000	5.65 × 10^−2^
Cobbles	100000	2.83 × 10^−2^
	200000	1.41 × 10^−2^

The difference in the K concentration for the Europan and the terrestrial ocean had an important outcome. Figure 3 shows that a 10 times greater concentration of K can provide enough sulfate for a 1000-fold increase in cell number. Table S2 shows that if we consider 1 kg of rocky material with an aqueous medium as small as 2 ml, as in the samples in the experimental work of water radiolysis^17^, scenarios *b* and *c* (described on section 4) significantly exceed the necessity to maintain a cell density of 4 × 10^7^ cells per liter, which is the average density that was present in samples of fracture water from the Witwatersrand basin region^17^.

Once more information regarding the existence, concentration and granulometry of pyrite and the presence of radioactive isotopes on the seabed of Europa is obtained from models, experiments or direct/indirect measurements by space missions, the results presented in Fig. 3 may be useful to estimate the habitability of the moon in terms of biologically useful energy sources.

## Discussion

In this work, deep terrestrial environments, such as the Mponeng gold mine, and selected Martian geological settings were evaluated as reasonable analogues for the under-crust oceanic non-illuminated and non-photosynthetic environment of Europa. In this context, the *Candidatus Desulforudis audaxviator* extremophile was used as a model organism because it is prominent from fracture water sampled from depths greater than 1.5 km across the Witwatersrand basin and it dominates the biota discovered 2.8 km below the land surface. The environmental conditions of Mponeng mine as a deep subsurface environment considering the lack of O_2_ and high temperatures can be considered similar to those of the seabed of Europa^37^, which is heated by tidal interaction with Jupiter. Because it is chemoautotrophic, this bacterium candidate species has the capacity to fix its own carbon (thus reducing the necessity of reduced organics) and to thrive in regions with a chemical disequilibrium produced by water radiolysis.

Our calculation assumes that there is enough radiolytic endogenous sulfate production to enter the microorganism metabolism even if we restrict the results to gamma-ray radiolysis. This simplification was based on the fact that the rate of radiolytic production of $H{O}^{\cdot }\,$from water, which reacts with pyrite to form sulfate, is at least one order of magnitude higher than the rate due to other decay channels, such as alphas (per electron-Volt)^32^. Despite the fact that rock porosity and space constraints were not considered in our calculations, those parameters were included in models to calculate the production far from the solid-water interface of radioactive mineral^28,38^. Here, a simple model considered local radiolysis in the aqueous environment, and this model showed enough to provide some reference to the objectives established for this work. In addition, considering the possibility for existence of hydrothermal systems on the Europa seabed^11^, these can be another endogenous source of sulfate^39^. These sources were assumed to exist far from our radiolytic system, and for the survival of a species such as *Ca. D. audaxviator*, it is necessary that the environment is depleted of oxygen and has a high pH^20^ – a condition normally not matched by nearby hydrothermal vents present on Earth^39^. More observational data from space missions are needed to constrain this information for the case of Europa. It has also been proposed that sulfate could have an exogenous source^3,40^, namely, from Io^40^. We argue that although this could be a source, the icy crust would prevent efficient mixing, made even less probable when associated with the abyssal depths of the ocean. It was assumed that sulfate is not used in other reactions that could prevent its availability for the microorganism. The main route for the depletion of sulfate could be, under Europan conditions, the precipitation of sulfate in the form of non-soluble salts such as BaSO_4_ and/or CaSO_4_. However, to study these possible sinks, more information is needed regarding the abundances of Ca^+^ and Ba^+^ and their equilibrium reactions under Europan conditions, especially pH and temperature. Additionally, reactions with silica can occur depending on the temperature, for example,$\,2CaS{O}_{4}+2Si{O}_{2}+C\to 2CaSi{O}_{3}+2S{O}_{2}+C{O}_{2}$, releasing sulfur in the form of gas. Thus, as the dynamics of cryotectonism is not completely understood and the rate of sulfate delivery or sinking remains poorly constrained, these effects were not taken into account in the present work. Future direct measurements and models are still needed to better constrain the habitability of Europa.

Other open questions arise, such as the unknown distribution of ^40^K on the ocean. If it is uniformly distributed, then the release of O_2_ by the radiolysis of water would have oxidized the ocean over time, which could halt sulfate production. However, a non-uniform distribution would place some restrictions on the present results, and although it is easy to imagine niches of uranium and thorium, it is difficult to imagine that there would also be a clump of ^40^K together with the niches of ^238^U and ^232^Th. An example of this clumping would be due to the presence of potassium minerals with low solubility such as jarosite (KFe_3_(OH)_6_(SO_4_)_2_).

Another product of radiolysis is H_2_O_2_, which is formed according to the reaction $H{O}^{\cdot }+H{O}^{\cdot }\to {H}_{2}{O}_{2}$. The sterilizing power of the peroxide could be a caveat. Too much peroxide near the microorganisms could minimize the habitability of the environment when considering the case of *Ca. D. audaxviator*, whose genes lack functional peroxidase homologs^20^. However, we suppose that this situation would not occur in a system such as the one modeled here, since H_2_O_2_ is a kinetically unstable chemical species and the rate of water formation from the same reagent $H{O}^{\cdot }$ is double the hydrogen peroxide formation rate^16,17^. Similarly, the presence of O_2_ as a product of radiolysis could be important considering its sterilizing power for *Ca. D. audaxviator*, as it also lacks a complete system for oxygen resistance^20^. However, deep environments such as the Mponeng gold mine and others are depleted of O_2_ and are reductant-rich^19,20,22,23^, which suggests that molecular oxygen and H_2_O_2_ have a relevant sink that may be the pyrite mineral itself, and the same may occur for the deep subsurface of Europa.

The results for the Europan framework are also useful for studies involving other Solar System icy moons that present similar geological activity and planetary formation history, such as Enceladus^30,41^. The Cassini mission showed a local chemical disequilibrium and evidence of the existence of hydrothermal systems as well as possible radiolysis under the icy shell of Enceladus^13^. In addition, like Europa, it is possible that Enceladus hosts minerals such as pyrite^30,42^. Thus, this moon is also a propitious celestial body to host life^43^. In other words, the habitability question addressed here could provide an analogue application for Enceladus, which is another promising target for astrobiology studies.

## Conclusions

Our results contribute to the evolving picture of Europa and other icy moons, such as Enceladus, as promising habitable environments. Sulfate production via γ-ray radiolysis was shown to be enough to supply the minimum energy required to maintain a considerable cell mass of the sulfate-reducing bacterium *Ca. D. audaxviator* used as a model organism. The cell quantity was shown to be comparable to that found in deep terrestrial environments if one assumes conditions similar to that in experimental work on radiolysis-produced sulfate. The total absence of sulfate sinks that could compete with a bacterial single-species ecosystem was assumed. However, as uranium also decays by α and β decay, the released energy should be greater than that calculated here, more water radiolysis would occur, and more pyrite would suffer oxidation. Therefore, this result can represent a lower limit for our sulfate-dependent ecosystem energy requirement. Otherwise, there would be no bacterial activity living in a subsurface environment such as that of the Mponeng gold mine. In fact, these and other deep and inhabited environments on Earth represent good analogues for Europa and could be further explored for this application, including serving as the basis for future space missions.

Our model for Europa can provide more energy than necessary to sustain the modeled microbial life even only by the gamma decay of ^40^K, since its abundance can be 10 times (or more) greater than that found in Earth’s oceans. This result makes Europa a propitious place for the development of an ecosystem that sustains forms of life such as the sulfate-reducing bacteria *Candidatus Desulforudis audaxviator*, although this estimate needs more constraints from experimental data. An important observation based on our results may be the relevance of the ^40^K concentration in the Europan ocean. As shown in Fig. 1, it creates a considerable difference in the range of the cell-carrying capacity based on the sulfate metabolism. The same dependency exists with pyrite grain size, and direct or indirect measurements of this characteristic are important to better constrain the model.

## Electronic supplementary material

Supplementary Information

## References

[1] Rothschild LJ, Mancinelli RL (2001). Life in extreme environments. Nature.

[2] Russell MJ (2014). The Drive to Life on Wet and Icy Worlds. Astrobiology.

[3] Hand KP, Carlson RW, Chyba CF (2007). Energy, Chemical Disequilibrium, and Geological Constraints on Europa. Astrobiology.

[4] Blair CC, D’Hondt S, Spivack AJ, Kingsley RH (2007). Radiolytic hydrogen and microbial respiration in subsurface sediments. Astrobiology.

[5] Cockell CS (2016). Habitability: A Review. Astrobiology.

[6] Vance S (2007). Hydrothermal systems in small ocean planets. Astrobiology.

[7] Hoehler TM (2007). An Energy Balance Concept for Habitability. Astrobiology.

[8] Chyba CF (2001). PLANETARY SCIENCE: Enhanced: Life Without Photosynthesis. Science (80-.)..

[9] Zahnle K, Schenk P, Levison H, Dones L (2003). Cratering rates in the outer solar system. Icarus.

[10] Grasset O (2013). JUpiter ICy moons Explorer (JUICE): An ESA mission to orbit Ganymede and to characterise the Jupiter system. Planet. Space Sci..

[11] Kargel J (2000). Europa’s Crust and Ocean: Origin, Composition, and the Prospects for Life. Icarus.

[12] McKay CP, Anbar AD, Porco C, Tsou P (2014). Follow the Plume: The Habitability of Enceladus. Astrobiology.

[13] Waite, J. H. *et al*. Cassini finds molecular hydrogen in the Enceladus plume: Evidence for hydrothermal processes. **356**, 155–159 (2017). 10.1126/science.aai8703 28408597

[14] Draganic, I. G. & Draganic, Z. D. The Radiation Chemistry of Water. **26** (1971).

[15] Draganic, I. G. Radiolysis of water: a look at its origin and occurrence in the nature. **72**, 181–186 (2005).

[16] Pastina, B. & Laverne, J. A. Effect of Molecular Hydrogen on Hydrogen Peroxide in Water Radiolysis. 9316–9322 (2001).

[17] Lefticariu L, Pratt LA, LaVerne JA, Schimmelmann A (2010). Anoxic pyrite oxidation by water radiolysis products - A potential source of biosustaining energy. Earth Planet. Sci. Lett..

[18] Draganic IG (1983). 1983. Natural nuclear reactors and ionizing radiation in the Precambrian. Precambrian Res., 20: 283–298..

[19] Lin LH (2005). Radiolytic H2 in continental crust: Nuclear power for deep subsurface microbial communities. Geochemistry, Geophys. Geosystems.

[20] Chivian D (2008). Environmental Genomics Reveals a Single-Species Ecosystem Deep Within Earth. Science (80-.)..

[21] Lin L-H (2006). Long-Term Sustainability of a High-Energy, Low-Diversity Crustal Biome. Science (80-.)..

[22] Dubessy, J. *et al*. Radiolysis evidenced by Hz-O2 and Hz-bearing fluid inclusions in three uranium deposits. **52** (1988).

[23] Savary V, Pagel M (1997). The effects of water radiolysis on local redox conditions in the Oklo, Gabon, natural fission reactors 10 and 16. Geochim. Cosmochim. Acta.

[24] Atri, D. On the possibility of galactic cosmic ray-induced radiolysis-powered life in subsurface environments in the Universe. *J. R. Soc. Interface* **13**, 20160459 (2016). 10.1098/rsif.2016.0459 PMC5095211 27707907

[25] Melott AL (2017). A Possible Role for Stochastic Astrophysical Ionizing Radiation Events in the Systematic Disparity between Molecular and Fossil Dates. Astrobiology.

[26] Marinho F, Paulucci L, Galante D (2014). Propagation and energy deposition of cosmic rays’ muons on terrestrial environments. Int. J. Astrobiol..

[27] Bouquet A, Glein CR, Wyrick D, Waite JH (2017). Alternative Energy: Production of H _2_ by Radiolysis of Water in the Rocky Cores of Icy Bodies. Astrophys. J..

[28] Dzaugis ME (2016). Radiolytic Hydrogen Production in the Subseafloor Basaltic Aquifer..

[29] Onstott TC (1997). Deep gold mines of South Africa: windows into the subsurface biosphere. Proc. SPIE.

[30] Sohl F (2010). Subsurface water oceans on icy satellites: Chemical composition and exchange processes. Space Sci. Rev..

[31] Hazen RM (2008). Mineral evolution. Am. Mineral..

[32] Pastina B, LaVerne JA (2001). Effect of Molecular Hydrogen on Hydrogen Peroxide in Water Radiolysis. J. Phys. Chem. A.

[33] Le Caër S (2011). Water Radiolysis: Influence of Oxide Surfaces on H2 Production under Ionizing Radiation. Water.

[34] Draganić IG, Bjergbakke E, Draganić ZD, Sehested K (1991). Decomposition of ocean waters by potassium-40 radiation 3800 Ma ago as a source of oxygen and oxidizing species. Precambrian Res..

[35] Lide, D. R. CRC Handbook of Chemistry and Physics. *eBook* 3485 978-1466571143 (2003).

[36] Wentworth CK (1922). A scale of grade and class terms for clastic sediments. J. Geol..

[37] Marion GM, Fritsen CH, Eicken H, Payne MC (2003). Factors, Potential Habitats, and Earth Analogues. Astrobiology.

[38] Dzaugis ME, Spivack AJ, Hondt SD (2015). A quantitative model of water radiolysis and chemical production rates near radionuclide-containing solids x. Radiat. Phys. Chem..

[39] Russell MJ, Arndt NT (2005). Geodynamic and metabolic cycles in the Hadean. Biogeosciences Discuss..

[40] Pasek Ma, Greenberg R (2012). Acidification of Europa’s subsurface ocean as a consequence of oxidant delivery. Astrobiology.

[41] Čadek O (2016). Enceladus’s internal ocean and ice shell constrained from Cassini gravity, shape, and libration data. Geophys. Res. Lett..

[42] Zolotov MY (2007). An oceanic composition on early and today’s Enceladus. Geophys. Res. Lett..

[43] McKay CP, Porco CC, Altheide T, Davis WL, Kral TA (2008). The Possible Origin and Persistence of Life on Enceladus and Detection of Biomarkers in the Plume. Astrobiology.

