# Author Correction: Microbial habitability of Europa sustained by radioactive sources

**DOI:** 10.1038/s41598-020-69041-8

**Published:** 2020-07-30

**Authors:** Thiago Altair, Marcio G. B. de Avellar, Fabio Rodrigues, Douglas Galante

**Affiliations:** 1 Brazilian Synchrotron Light Laboratory (LNLS), Brazilian Center for Research in Energy and Materials (CNPEM), Av. Giuseppe Máximo Scolfaro, 10000, 13083-100 Campinas, SP Brazil; 2 Instituto de Astronomia, Geofísica E Ciências Atmosféricas, Universidade de São Paulo, Rua Do Matão, 1226, 05508-090 São Paulo, SP Brazil; 3 Departamento de Química Fundamental Instituto de Química, Universidade de São Paulo, Av. Prof. Lineu Prestes, 748, 05508-000 São Paulo, SP Brazil; 4 Programa de Pós-Graduação Em Física Biomolecular, Instituto de Física de São Carlos, Universidade de São Paulo, São Carlos, SP Brazil

Correction to: *Scientific reports* 10.1038/s41598-017-18470-z, published online 10 January 2018

This Article contains errors.

In the Results,

“The difference in the K concentration for the Europan and the terrestrial ocean had an important outcome. Figure 3 shows that a 10 times greater concentration of K can provide enough sulfate for a 1000-fold increase in cell number. Table S2 shows that if we consider 1 kg of rocky material with an aqueous medium as small as 2 ml, as in the samples in the experimental work of water radiolysis^17^, scenarios b and c (described on Section 4) significantly exceed the necessity to maintain a cell density of 4 × 10^7^ cells per liter, which is the average density that was present in samples of fracture water from the Witwatersrand basin region^17^”

Should read:

“The difference in the K concentration for the Europan and the terrestrial ocean has significative outcome only on scenario a, as shown in Figure 3. On other scenarios, the results for different K concentration have overlapped each other. Table S2 shows that if we consider 1 kg of rocky material with an aqueous medium as small as 2 ml, as in the samples in the experimental work of water radiolysis^17^, scenarios b and c (described on Section 4) significantly exceed the number of cell density of 4 × 10^7^ cells per liter, which is the average density that was present in samples of fracture water from the Witwatersrand basin region^17^”

Additionally, in Figure 3 the Log-Log plot for scenario b and c were incorrectly presented and shows the minimum and maximum potassium concentrations. The correct Figure 3 appears below as Figure 1. As a result, the Figure legend,

“Log–Log plot of the cell-carrying capacity per mass of rocks that contains pyrite compared to the results for the different uranium and thorium scenarios (a, b and c, as described in Section 4) and the assumed minimum (light gray) and maximum (dark gray) potassium concentrations. The X-axis represents the variation in grain size of pyrite based on the classification and based on the Wentworth scale (see Table S2), which is inversely proportional to the surface area available for oxidation.”

Should read:

“Fig. 3—Log–Log plot of the cell-carrying capacity per mass of rocks that contains pyrite compared to the results for the different uranium and thorium scenarios (a, b and c, as described in Section 4) and the assumed minimum (light gray) and maximum (dark gray) potassium concentrations. For scenario b and c, results related to different K concentration have overlapped. The X-axis represents the variation in grain size of pyrite based on the classification and based on the Wentworth scale (see Table S2), which is inversely proportional to the surface area available for oxidation.”

**Figure 1:**
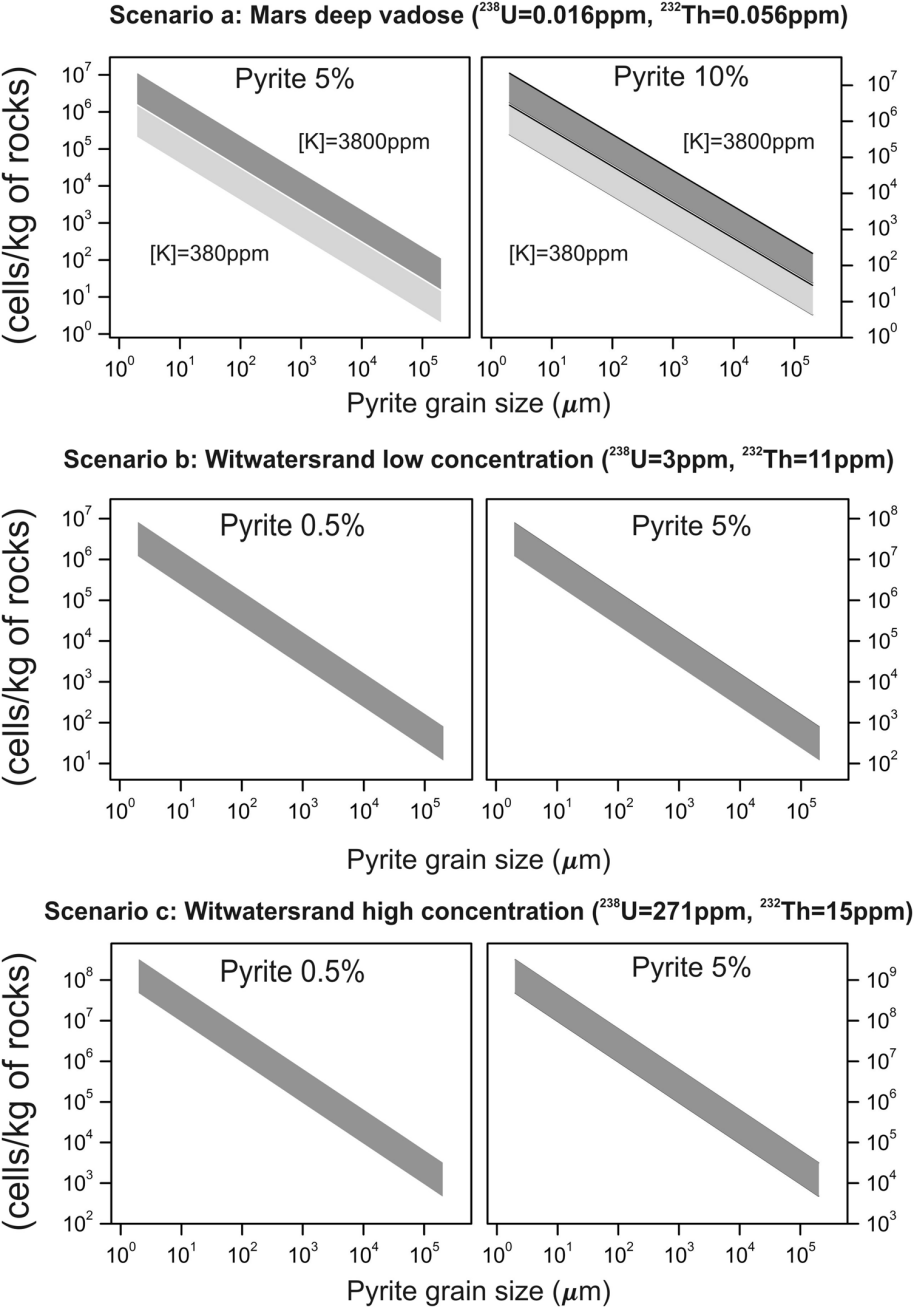
Log-Log plot of the cell-carrying capacity per mass of rocks that contains pyrite compared to the results for the different uranium and thorium scenarios (**a**, **b** and **c**, as described in Section 4) and the assumed minimum (light gray) and maximum (dark gray) potassium concentrations. For scenario b and c, results related to different K concentration have overlapped. The X-axis represents the variation in grain size of pyrite based on the classification and based on the Wentworth scale (see Table S2), which is inversely proportional to the surface area available for oxidation.

Finally, the Supplementary Information file that accompanies this Article contains errors in Supplementary Tables S2 and S3. These errors were caused by an incorrect variable used in the computer program which generated the sulphate production rate. The correct tables S2 and S3 appear below.

**Table S2:**
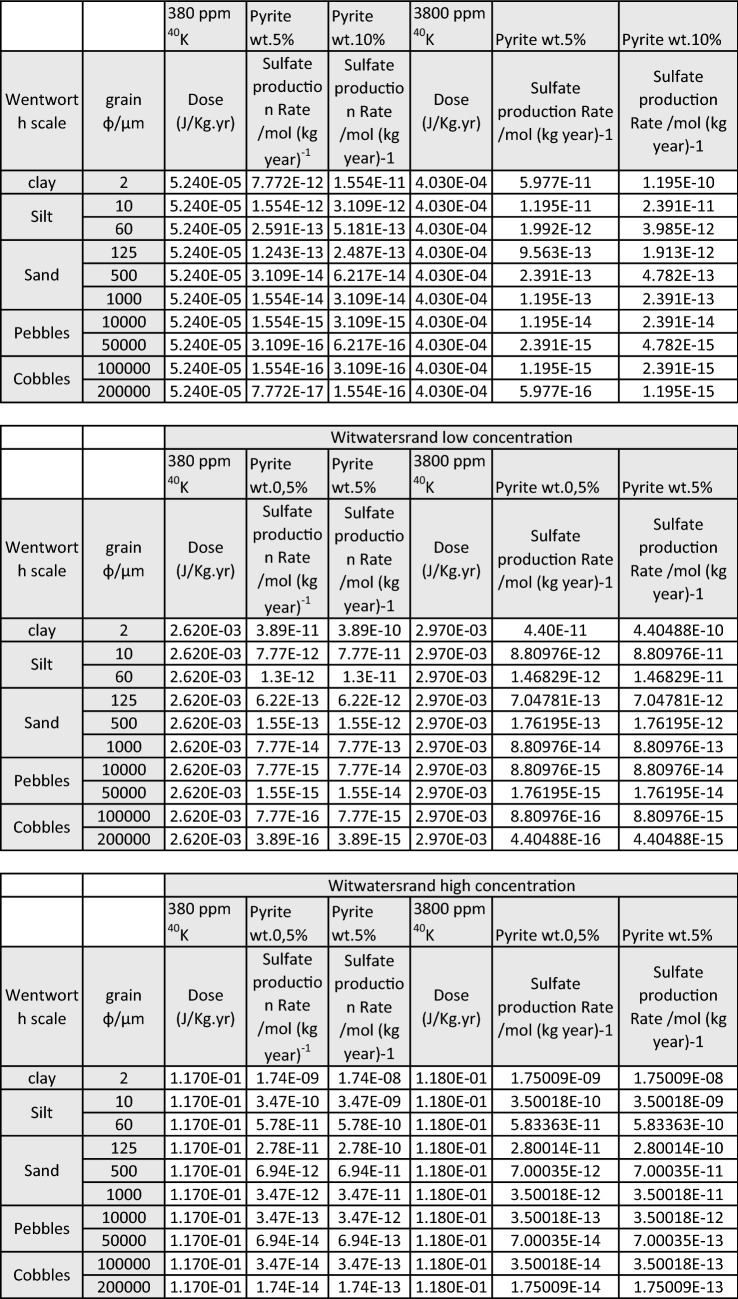
.

**Table S3:**
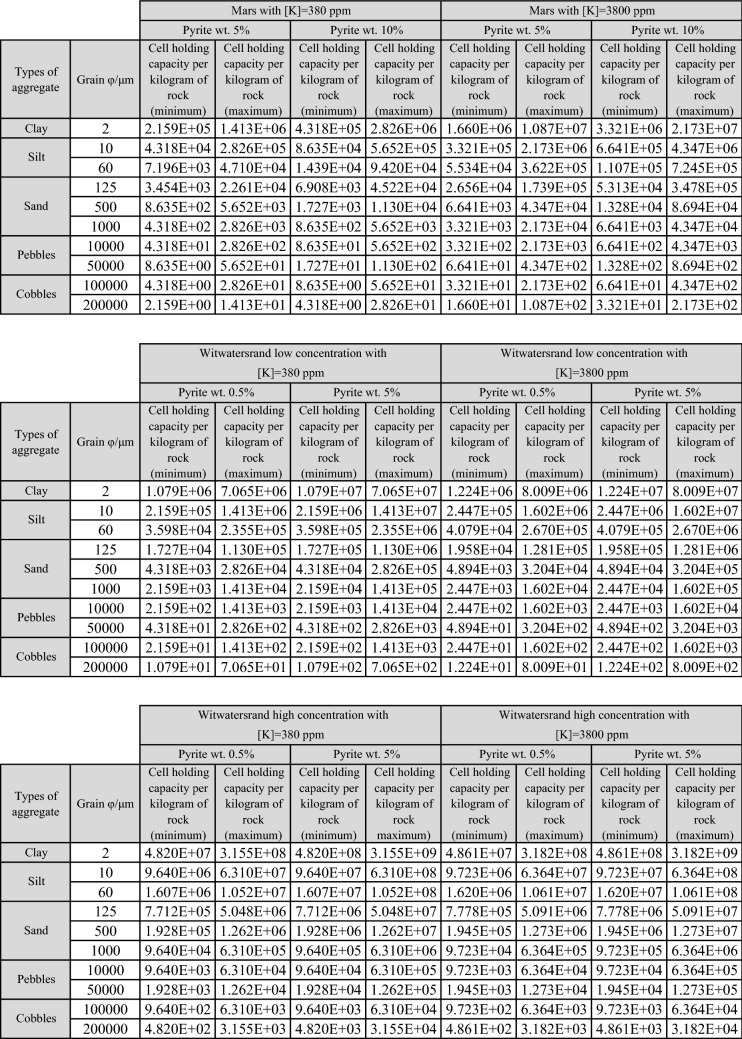
.

