# Supplementary material for: Microbial habitability of Europa sustained by radioactive sources
Source: Sci Rep. 2018 Jan 10;8:260. doi: 10.1038/s41598-017-18470-z (PMC5762670; doi:10.1038/s41598-017-18470-z)
Supplement: Supplementary file 1 — Supplementary Information [file 41598_2017_18470_MOESM1_ESM.doc]

Supplementary Online material for

**Microbial habitability of Europa sustained by radioactive sources**

Thiago Altair^1,4^, Marcio G. B. de Avellar^2^, Fabio Rodrigues^3^, Douglas Galante^1,4^

^1^ Brazilian Synchrotron Light Laboratory (LNLS), Brazilian Center for Research in Energy and Materials (CNPEM), Campinas/SP, Brazil.

^2^ Instituto de Astronomia, Geofísica e Ciências Atmosféricas, Universidade de São Paulo, São Paulo/SP, Brazil.

^3^ Departamento de Química Fundamental Instituto de Química, Universidade de São Paulo, São Paulo/SP, Brazil.

^4^ Programa de Pós-Graduação em Física Biomolecular, Instituto de Física de São Carlos, Universidade de São Paulo, São Carlos/SP, Brazil.

*Wentworth granular scale*

As a reference for the selected range of pyrite grain size for sulfate production, it is used the granular size classification proposed by Wentworth (1922). In this scheme, the names applied to the different grades carry no lithologic, mineralogic or chemical significance as far as the present work is concerned. The author considers that such uniformity in each sediment does not occur in nature, as on the example of clay particles that are not usually composed of the same material. Regardless of this and other possible caveats, the work that presents the classification was cited more than four thousand times until the submission of our paper, according to public databases of academic journals. This grain scale classification and these terms are now generally followed as a reference^1,2^. The scale is based on the geometric distribution of grain size extending from 1/256 to 256 mm. From its establishment until now, it was reviewed, adopting the so called φ-scale, which has a logarithm relationship with the grain size^2^. On the main manuscript, it was considered as just another expression for the grain size. This commonly adopted size scale is presented on figure S1.


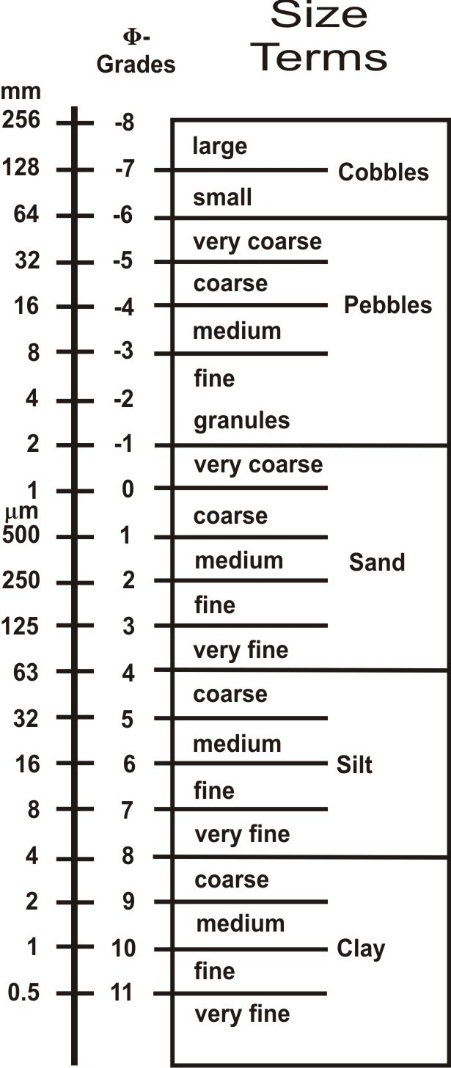


Figure S1- Wentworth grain-size scale and grain-size-based terms commonly used for differentiating sedimentary grains (adapted from Flügel 2010^2^).

As long that there is still a lack of detailed information or modelling about the possible presence of pyrite in Europa subsurface environment - which includes its granulation setting – it is reasonable to evaluate a range of possible granulations, based on the Wentworth scale.

*Sulfate production in each scenario proposed*

Based on Lefticariu et al. (2010), we proposed the calculations for sulfate production for each scenario proposed. However, to adequate the terrestrial and martian scenarios proposed as analogues for subsurface environments of Europa, we have considered not only ^238^U and ^232^Th, but also ^40^K. And, as mentioned in the main manuscript, we have proposed to calculate sulfate using the concentrations for ^238^U and ^232^Th constrained to each scenario, as suggested in the work of Lefticariu et al. and the ^40^K concentration suggested for terrestrial ocean^3^ and for an estimation for Europan ocean^4^. The results in detail for sulfate production are shown in table S1.

Table S1. Estimated radiolytic sulfate production for different proposed scenarios. Each scenario represents a local composition for ^238^U and ^232^Th. Concentration for each scenario: Mars deep vadose: ^238^U=0.016ppm, ^232^Th=0.056ppm; Witwatersrand with low concentration: ^238^U=3ppm, ^232^Th=11ppm; Witwatersrand with high concentration: ^238^U=271ppm , ^232^Th=15ppm. For each, it is considered a specific grain size of pyrite in the classification based on Wentworth scale and it is considered ^40^K concentration as 0.0117% of total potassium ^5^.

|  | | Mars deep vadose | | | | | |
| --- | --- | --- | --- | --- | --- | --- | --- |
|  |  | [K]=380ppm | | | [K]=3800ppm | | |
|  |  |  | Pyrite  wt. 5% | Pyrite  wt. 10% |  | Pyrite  wt. 5% | Pyrit  wt. 10% |
| Wentworth scale | Grain φ/μm | Dose (J/Kg.yr) | Sulfate production Rate /mol  (kg year)^-1^ | Sulfate production Rate /mol  (kg year)^-1^ | Dose (J/Kg.yr) | Sulfate production Rate /mol  (kg year)^-1^ | Sulfate production Rate /mol  (kg year)^-1^ |
| Clay | 2 | 5.24×10^-5^ | 7.77×10^-12^ | 1.55×10^-11^ | 4.03×10^-4^ | 1.20×10^-7^ | 2.39×10^-7^ |
| Silt | 10 | 5.24×10^-5^ | 1.55×10^-12^ | 3.11×10^-12^ | 4.03×10^-4^ | 2.39×10^-8^ | 4.78×10^-8^ |
|  | 60 | 5.24×10^-5^ | 2.59×10^-13^ | 5.18×10^-13^ | 4.03×10^-4^ | 3.99×10^-9^ | 7.97×10^-9^ |
| Sand | 125 | 5.24×10^-5^ | 1.24×10^-13^ | 2.49×10^-13^ | 4.03×10^-4^ | 1.91×10^-9^ | 3.83×10^-9^ |
|  | 500 | 5.24×10^-5^ | 3.11×10^-14^ | 6.22×10^-14^ | 4.03×10^-4^ | 4.78×10^-10^ | 9.57×10^-10^ |
|  | 1000 | 5.24×10^-5^ | 1.55×10^-14^ | 3.11×10^-14^ | 4.03×10^-4^ | 2.39×10^-10^ | 4.78×10^-10^ |
| Pebbles | 10000 | 5.24×10^-5^ | 1.55×10^-15^ | 3.11×10^-15^ | 4.03×10^-4^ | 2.39×10^-11^ | 4.78×10^-11^ |
|  | 50000 | 5.24×10^-5^ | 3.11×10^-16^ | 6.22×10^-16^ | 4.03×10^-4^ | 4.78×10^-12^ | 9.57×10^-12^ |
| Cobbles | 100000 | 5.24×10^-5^ | 1.55×10^-16^ | 3.11×10^-16^ | 4.03×10^-4^ | 2.39×10^-12^ | 4.78×10^-12^ |
|  | 200000 | 5.24×10^-5^ | 7.77×10^-17^ | 1.55×10^-16^ | 4.03×10^-4^ | 1.20×10^-12^ | 2.39×10^-12^ |
|  | | | | | | | |
|  | | Witwatersrand low concentration | | | | | |
|  |  | [K]=380ppm | | | [K]=3800ppm | | |
|  |  |  | Pyrite  wt. 0.5% |  | Pyrite  wt. 0.5% |  | Pyrite  wt. 0.5% |
| Wentworth scale | Grain φ/μm | Dose (J/Kg.yr) | Sulfate production Rate /mol (kg year)^-1^ | Sulfate production Rate /mol (kg year)^-1^ | Dose (J/Kg.yr) | Sulfate production Rate /mol (kg year)^-1^ | Sulfate production Rate /mol (kg year)^-1^ |
| Clay | 2 | 2.62×10^-3^ | 3.89×10^-10^ | 3.89×10^-10^ | 2.97×10^-3^ | 8.81×10^-7^ | 2.20×10^-5^ |
| Silt | 10 | 2.62×10^-3^ | 7.77×10^-11^ | 7.77×10^-11^ | 2.97×10^-3^ | 1.76×10^-7^ | 4.41×10^-6^ |
|  | 60 | 2.62×10^-3^ | 1.30×10^-11^ | 1.30×10^-11^ | 2.97×10^-3^ | 2.94×10^-8^ | 7.34×10^-7^ |
| Sand | 125 | 2.62×10^-3^ | 6.22×10^-12^ | 6.22×10^-12^ | 2.97×10^-3^ | 1.41×10^-8^ | 3.53×10^-7^ |
|  | 500 | 2.62×10^-3^ | 1.55×10^-12^ | 1.55×10^-12^ | 2.97×10^-3^ | 3.53×10^-9^ | 8.81×10^-8^ |
|  | 1000 | 2.62×10^-3^ | 7.77×10^-13^ | 7.77×10^-13^ | 2.97×10^-3^ | 1.76×10^-9^ | 4.41×10^-8^ |
| Pebbles | 10000 | 2.62×10^-3^ | 7.77×10^-14^ | 7.77×10^-14^ | 2.97×10^-3^ | 1.76×10^-10^ | 4.41×10^-9^ |
|  | 50000 | 2.62×10^-3^ | 1.55×10^-14^ | 1.55×10^-14^ | 2.97×10^-3^ | 3.53×10^-11^ | 8.81×10^-10^ |
| Cobbles | 100000 | 2.62×10^-3^ | 7.77×10^-15^ | 7.77×10^-15^ | 2.97×10^-3^ | 1.76×10^-11^ | 4.41×10^-10^ |
|  | 200000 | 2.62×10^-3^ | 3.89×10^-15^ | 3.89×10^-15^ | 2.97×10^-3^ | 8.81×10^-12^ | 2.20×10^-10^ |
|  |  |  |  |  |  |  |  |
|  | | Witwatersrand high concentration | | | | | |
|  |  | [K]=380ppm | | | [K]=3800ppm | | |
|  |  |  | Pyrite  wt. 0.5% | Pyrite  wt. 5% |  | Pyrite  wt. 0.5% | Pyrite  wt. 5% |
| Wentworth scale | Grain φ/μm | Dose (J/Kg.yr) | Sulfate production Rate /mol  (kg year)^-1^ | Sulfate production Rate /mol  (kg year)^-1^ | Dose (J/Kg.yr) | Sulfate production Rate /mol  (kg year)^-1^ | Sulfate production Rate /mol  (kg year)^-1^ |
| Clay | 2 | 1.17×10^-1^ | 1.74×10^-9^ | 1.74×10^-8^ | 1.18×10^-1^ | 8.75×10^-4^ | 3.91×10^-2^ |
| Silt | 10 | 1.17×10^-1^ | 3.47×10^-10^ | 3.47×10^-9^ | 1.18×10^-1^ | 1.75×10^-4^ | 7.82×10^-3^ |
|  | 60 | 1.17×10^-1^ | 5.78×10^-11^ | 5.78×10^-10^ | 1.18×10^-1^ | 2.92×10^-5^ | 1.30×10^-3^ |
| Sand | 125 | 1.17×10^-1^ | 2.78×10^-11^ | 2.78 ×10^-10^ | 1.18×10^-1^ | 1.40×10^-5^ | 6.25×10^-4^ |
|  | 500 | 1.17×10^-1^ | 6.94×10^-12^ | 6.94×10^-11^ | 1.18×10^-1^ | 3.50×10^-6^ | 1.56×10^-4^ |
|  | 1000 | 1.17×10^-1^ | 3.47×10^-12^ | 3.47×10^-11^ | 1.18×10^-1^ | 1.75×10^-6^ | 7.82×10^-5^ |
| Pebbles | 10000 | 1.17×10^-1^ | 3.47×10^-13^ | 3.47×10^-12^ | 1.18×10^-1^ | 1.75×10^-7^ | 7.82×10^-6^ |
|  | 50000 | 1.17×10^-1^ | 6.94×10^-14^ | 6.94×10^-13^ | 1.18×10^-1^ | 3.50×10^-8^ | 1.56×10^-6^ |
| Cobbles | 100000 | 1.17×10^-1^ | 3.47×10^-14^ | 3.47×10^-13^ | 1.18×10^-1^ | 1.75×10^-8^ | 7.82×10^-7^ |
|  | 200000 | 1.17×10^-1^ | 1.74×10^-14^ | 1.74×10^-13^ | 1.18×10^-1^ | 8.75×10^-9^ | 3.91×10^-7^ |

*Results for the cell holding capacity per kilogram of rock*

Table S2. Estimated cell holding capacity for *Ca. D. audaxviator* for different scenarios and considering different possible pyrite aggregates. Each scenario represents a local composition for ^238^U and ^232^Th. Concentration for each scenario: Mars deep vadose: ^238^U=0.016ppm, ^232^Th=0.056ppm; Witwatersrand with low concentration: ^238^U=3ppm , ^232^Th=11ppm ; Witwatersrand with high concentration: ^238^U=271ppm , ^232^Th=15ppm. For each, it is considered a specific grain size of pyrite in the classification based on Wentworth scale and it is considered ^40^K concentration as 0,0117% of total potassium ^5^.

|  | | Mars with [K]=380 ppm | | | | Mars with [K]=3800 ppm | | | |
| --- | --- | --- | --- | --- | --- | --- | --- | --- | --- |
|  |  | Pyrite wt. 5% | | Pyrite wt. 10% | | Pyrite wt. 5% | | Pyrite wt. 10% | |
| Types of aggregate | Grain φ/μm | Cell holding capacity per kilogram of rock (minimum) | Cell holding capacity per kilogram of rock (maximum) | Cell holding capacity per kilogram of rock (minimum) | Cell holding capacity per kilogram of rock (maximum) | Cell holding capacity per kilogram of rock (minimum) | Cell holding capacity per kilogram of rock (maximum) | Cell holding capacity per kilogram of rock (minimum) | Cell holding capacity per kilogram of rock (maximum) |
| Clay | 2 | 2.16×10^5^ | 1.41×10^6^ | 2.13×10^2^ | 1.40×10^3^ | 4.26×10^2^ | 2.79×10^3^ | 3.28×10^3^ | 2.15×10^4^ |
| Silt | 10 | 4.32×10^4^ | 2.83×10^5^ | 4.26×10^1^ | 2.79×10^2^ | 8.53×10^1^ | 5.58×10^2^ | 6.56×10^2^ | 4.29×10^3^ |
|  | 60 | 7.20×10^3^ | 4.71×10^4^ | 7.11 | 4.65×10^1^ | 1.42×10^1^ | 9.30×10^1^ | 1.09×10^2^ | 7.15×10^2^ |
| Sand | 125 | 3.45×10^3^ | 2.26×10^4^ | 3.41 | 2.23×10^1^ | 6.82 | 4.46×10^1^ | 5.25×10^1^ | 3.43×10^2^ |
|  | 500 | 8.64×10^2^ | 5.65×10^3^ | 8.53×10^-1^ | 5.58 | 1.71 | 1.12×10^1^ | 1.31×10^1^ | 8.58×10^1^ |
|  | 1000 | 4.32×10^2^ | 2.83×10^3^ | 4.26×10^-1^ | 2.79 | 8.53×10^-1^ | 5.58 | 6.56 | 4.29×10^1^ |
| Pebbles | 10000 | 4.32×10^1^ | 2.83×10^2^ | 4.26×10^-2^ | 2.79×10^-1^ | 8.53×10^-2^ | 5.58×10^-1^ | 6.56×10^-1^ | 4.29 |
|  | 50000 | 8.64×10 | 5.65×10^1^ | 8.53×10^-3^ | 5.58×10^-2^ | 1.71×10^-2^ | 1.12×10^-1^ | 1.31×10^-1^ | 8.58×10^-1^ |
| Cobbles | 100000 | 4.32 | 2.83×10^1^ | 4.26×10^-3^ | 2.79×10^-2^ | 8.53×10^-3^ | 5.58×10^-2^ | 6.56×10^-2^ | 4.29×10^-1^ |
|  | 200000 | 2.16 | 1.41×10^1^ | 2.13×10^-3^ | 1.40×10^-2^ | 4.26×10^-3^ | 2.79×10^-2^ | 3.28×10^-2^ | 2.15×10^-1^ |

|  | | Witwatersrand low concentration with  [K]=380 ppm | | | | Witwatersrand low concentration with  [K]=3800 ppm | | | |
| --- | --- | --- | --- | --- | --- | --- | --- | --- | --- |
|  |  | Pyrite wt. 5% | | Pyrite wt. 10% | | Pyrite wt. 5% | | Pyrite wt. 10% | |
| Types of aggregate | Grain φ/μm | Cell holding capacity per kilogram of rock (minimum) | Cell holding capacity per kilogram of rock (maximum) | Cell holding capacity per kilogram of rock (minimum) | Cell holding capacity per kilogram of rock (maximum) | Cell holding capacity per kilogram of rock (minimum) | Cell holding capacity per kilogram of rock (maximum) | Cell holding capacity per kilogram of rock (minimum) | Cell holding capacity per kilogram of rock (maximum) |
| Clay | 2 | 1.02×10^4^ | 6.70×10^4^ | 1.02×10^4^ | 6.70×10^4^ | 2.42×10^7^ | 1.58×10^8^ | 5.80×10^8^ | 3.80×10^9^ |
| Silt | 10 | 2.05×10^3^ | 1.34×10^4^ | 2.05×10^3^ | 1.34×10^4^ | 4.83×10^6^ | 3.16×10^7^ | 1.16×10^8^ | 7.60×10^8^ |
|  | 60 | 3.41×10^2^ | 2.23×10^3^ | 3.41×10^2^ | 2.23×10^3^ | 8.06×10^5^ | 5.27×10^6^ | 1.93×10^7^ | 1.27×10^8^ |
| Sand | 125 | 1.64×10^2^ | 1.07×10^3^ | 1.64×10^2^ | 1.07×10^3^ | 3.87×10^5^ | 2.53×10^6^ | 9.29×10^6^ | 6.08×10^7^ |
|  | 500 | 4.09×10^1^ | 2.68×10^2^ | 4.09×10^1^ | 2.68×10^2^ | 9.67×10^4^ | 6.33×10^5^ | 2.32×10^6^ | 1.52×10^7^ |
|  | 1000 | 2.05×10^1^ | 1.34×10^2^ | 2.05×10^1^ | 1.34×10^2^ | 4.83×10^4^ | 3.16×10^5^ | 1.16×10^6^ | 7.60×10^6^ |
| Pebbles | 10000 | 2.05 | 1.34×10^1^ | 2.05 | 1.34×10^1^ | 4.83×10^3^ | 3.16×10^4^ | 1.16×10^5^ | 7.60×10^5^ |
|  | 50000 | 4.09×10^-1^ | 2.68 | 4.09×10^-1^ | 2.68 | 9.67×10^2^ | 6.33×10^3^ | 2.32×10^4^ | 1.52×10^5^ |
| Cobbles | 100000 | 2.05×10^-1^ | 1.34 | 2.05×10^-1^ | 1.34 | 4.83×10^2^ | 3.16×10^3^ | 1.16×10^4^ | 7.60×10^4^ |
|  | 200000 | 1.02×10^-1^ | 6.70×10^-1^ | 1.02×10^-1^ | 6.70×10^-1^ | 2.42×10^2^ | 1.58×10^3^ | 5.80×10^3^ | 3.80×10^4^ |

|  | | Witwatersrand high concentration with  [K]=380 ppm | | | | Witwatersrand high concentration with  [K]=3800 ppm | | | |
| --- | --- | --- | --- | --- | --- | --- | --- | --- | --- |
|  |  | Pyrite wt. 5% | | Pyrite wt. 10% | | Pyrite wt. 5% | | Pyrite wt. 10% | |
| Types of aggregate | Grain φ/μm | Cell holding capacity per kilogram of rock (minimum) | Cell holding capacity per kilogram of rock (maximum) | Cell holding capacity per kilogram of rock (minimum) | Cell holding capacity per kilogram of rock maximum) | Cell holding capacity per kilogram of rock (minimum) | Cell holding capacity per kilogram of rock (maximum) | Cell holding capacity per kilogram of rock (minimum) | Cell holding capacity per kilogram of rock (maximum) |
| Clay | 2 | 4.82×10^7^ | 4.33×10^4^ | 2.83×10^5^ | 4.33×10^5^ | 2.83×10^6^ | 5.54×10^5^ | 3.63×10^6^ | 2.34×10^7^ |
| Silt | 10 | 9.64×10^6^ | 8.66×10^3^ | 5.67×10^4^ | 8.66×10^4^ | 5.67×10^5^ | 1.11×10^5^ | 7.25×10^5^ | 4.69×10^6^ |
|  | 60 | 1.61×10^6^ | 1.44×10^3^ | 9.44×10^3^ | 1.44×10^4^ | 9.44×10^4^ | 1.85×10^4^ | 1.21×10^5^ | 7.81×10^5^ |
| Sand | 125 | 7.71×10^5^ | 6.92×10^2^ | 4.53×10^3^ | 6.92×10^3^ | 4.53×10^4^ | 8.87×10^3^ | 5.80×10^4^ | 3.75×10^5^ |
|  | 500 | 1.93×10^5^ | 1.73×10^2^ | 1.13×10^3^ | 1.73×10^3^ | 1.13×10^4^ | 2.22×10^3^ | 1.45×10^4^ | 9.37×10^4^ |
|  | 1000 | 9.64×10^4^ | 8.66×10^1^ | 5.67×10^2^ | 8.66×10^2^ | 5.67×10^3^ | 1.11×10^3^ | 7.25×10^3^ | 4.69×10^4^ |
| Pebbles | 10000 | 9.64×10^3^ | 8.66 | 5.67×10^1^ | 8.66×10^1^ | 5.67×10^2^ | 1.11×10^2^ | 7.25×10^2^ | 4.69×10^3^ |
|  | 50000 | 1.93×10^3^ | 1.73 | 1.13×10^1^ | 1.73×10^1^ | 1.13×10^2^ | 2.22×10^1^ | 1.45×10^2^ | 9.37×10^2^ |
| Cobbles | 100000 | 9.64×10^2^ | 8.66×10^-1^ | 5.67 | 8.66 | 5.67×10^1^ | 1.11×10^1^ | 7.25×10^1^ | 4.69×10^2^ |
|  | 200000 | 4.82×10^2^ | 4.33×10^-1^ | 2.83 | 4.33 | 2.83×10^1^ | 5.54 | 3.63×10^1^ | 2.34×10^2^ |

Figure 3 on the main manuscript illustrates the result for our calculation of the cell holding capacity as on Table S2 for each proposed scenario. For Figure 3, we have plotted these results in a log-log scale graph, once the results in normal scale showed an exponential-like distribution. We have highlighted the region between the maximum and minimum for the cell holding capacity per kilogram of rock to illustrate the range of the results for each scenario.

**References**

1. Folk, R. L. A REVIEW OF GRAIN-SIZE PARAMETERS. *Sedimentology* **6,** 73–93 (1966).

2. Flügel, E. *Microfacies of Carbonate Rocks*. *Climate Change 2013 - The Physical Science Basis* **1,** (Springer Berlin Heidelberg, 2010).

3. Stumm, W. & Morgan, J. J. *Aquatic Chemistry*. (Wiley, 1981).

4. Chyba, C. F. PLANETARY SCIENCE: Enhanced: Life Without Photosynthesis. *Science (80-. ).* **292,** 2026–2027 (2001).

5. Lide, D. R. CRC Handbook of Chemistry and Physics. *eBook* 3485 (2003). doi:978-1466571143
